# Coexistence Between Antiphospholipid Syndrome and Protein S Deficiency in a Patient With Transverse Sinus Thrombosis: A Rare Association

**DOI:** 10.1002/ccr3.9663

**Published:** 2024-12-05

**Authors:** Ziryab Imad Taha Mahmoud, Yassin Abdelrahim Abdalla, Hoyam Bakri Gafar Elhaj Omer, Obada Mohamed Ahmed Ali, Muhammad Salah Ali Suliman, Asma Awad, Salih Boushra Hamza, Sara Omar Elamin Elmobark, Mohammedelmuntaga Gafar, Abuobieda Omer Osman

**Affiliations:** ^1^ University of Bahri Khartoum Sudan; ^2^ Ziryab Research Group Khartoum Sudan; ^3^ Internal Medicine Department Omdurman Islamic University Khartoum Sudan; ^4^ Internal Medicine Department National Ribat University Khartoum Sudan; ^5^ Internal Medicine Department Cairo University Cairo Egypt; ^6^ Internal Medicine Department Khartoum University Khartoum Sudan; ^7^ Internal Medicine Department Alzaiem Alazhari University Khartoum Sudan; ^8^ Department of Radiology Sudanese Medical Specialization Board Khartoum Sudan

**Keywords:** antiphospholipid syndrome, case reports, protein S, sinus thrombosis

## Abstract

The primary antiphospholipid syndrome and protein S deficiency are known hypercoagulable states predisposing to strokes. We present a 34‐year‐old woman presented to rheumatology clinic complaining of right side weakness and aphasia for 2 months before the visit. There was joint pain in the right elbow and shoulder joints, hyperpigmentation on her face and dry painful red eyes mainly the right eye in addition to dry mouth. She had a history of recurrent abortions. Neurological examination showed hypertonia on right lower and upper limb and normal on left one, while the power was grade 3 on right side and normal in left one. Upper motor neuron signs and facial palsy was noted. Hyperpigmentation in the face was observed. MRI brain showed that left temporoparietal hemorrhagic infraction involving the basal ganglia and MRV brain showed left transverse sinus thrombosis and attenuation of sigmoid and internal jugular vein. B2‐glycoprotein Ig AGM, lupus anticoagulant, anticardiolipin (IgG, IgM, and IgA), protein S were positive. ANA profile was borderline for PCNA. We report unusual venous sinus thrombosis with primary antiphospholipid syndrome and acquired protein S deficiency.

**Summary**

- Coexistence between APS and protein S deficiency is an uncommon possible cause of CVT.
- Those who were lupus anticoagulant positive and anticardiolipin antibody negative were found to develop free protein S deficiency; probably due to the abnormal binding site of free protein S to the C4b‐BP.
- Diagnosis of APS in background of CVST is crucial for effective management, as CVST may recur again in suboptimal management.

## 1 Introduction

Thrombophilia can be defined as an increased tendency to develop a thrombus. Thrombophilia etiology divides into either heritable defects, such as mutations in the genes encoding the natural anticoagulants antithrombin, protein C, protein S, clotting factors, prothrombin, and factor V, or acquired defects, such as antiphospholipid syndrome (APS) [1].

APS is an autoimmune disease characterized by the presence of antiphospholipid antibodies, such as lupus anticoagulant, anticardiolipin antibodies, and anti‐β2‐glycoprotein [1]. APS can present with a variety of clinical phenotypes, including arterial, venous thrombosis, and obstetric complications [2].

Certain projections suggest that the occurrence of APS stands at approximately 5 fresh instances per year for every 100,000 individuals, with a prevalence of roughly 40–50 cases per 100,000 people [3]. A study done in 1990 found that in a series of 51 unselected patients presenting with stroke and transient ischemic attacks, three had APS [4].

Diagnosis of APS is based on at least one clinical manifestation thrombosis and pregnancy morbidity beside persistently positive antiphospholipid antibodies.

Patients with APS may have diverse neurological condition these include cerebrovascular accident, epilepsy, cognitive disorders, headaches/migraine, chorea, multiple sclerosis‐like, transverse myelitis, ocular symptoms, and Guillain–Barré syndrome [5].

Another cause of thrombophilia is protein C and S deficiency. Protein C and protein S are glycoproteins, predominantly synthesized in the liver, that are important components of the natural anticoagulation system in the body. They are vitamin K‐dependent and serve as essential components in the maintenance of physiologic hemostasis [1].

Individuals with inherited abnormalities in the protein C and protein S pathways have an increased susceptibility to thromboembolic occurrences like deep vein thrombosis, pulmonary embolism, stroke, and organ ischemia [6].

Cerebral venous thrombosis (CVT) is considered as a rare cause of stroke (represent 1% of all stroke form in adult) [1]. It tends to affect females which may he attributed to some gender‐associated risks as pregnancy and oral contraceptive use [2].

Due to its variable presentation; the diagnosis of CVT is still challenging. However, there are common clinical manifestations which include headache, seizure, focal neurological deficit, and altered sensorium. These symptoms may evolve over several days. Risk factors of CVT include hereditary thrombophilia, autoimmune disorder, malignancy and chronic inflammatory diseases. It is worth mentioning that patients with CVT may have more than one risk factor [3].

Venous sinus thrombosis can be seen, with or without other peripheral thrombotic events, especially in patients with APS [7].

In study carried out in 624 patients with CVT APS was the underlying cause in 5.9% [8]. Although occurrence of CVT in background of APS is relatively uncommon (7 out of 1000 APS patients has been reported), diagnosis of it is essential because it need a different management approach and follow‐up [9, 10].

## 2 Case Presentation

### 2.1 Patient History

A 34‐year‐old female smoker with free medical background was referred to our rheumatology clinic after she had been discharged after admission due to an ischemic stroke. The attack was described as weakness on the right side with deviation of the mouth to the left side, accompanied by central loss of vision. On further questioning, the patient described joint pain in the right elbow and shoulder and dryness in her mouth and eyes. Past medical history is significant for multiple abortions and the menstrual cycle's irregularities. No history of contraceptive use or long‐term medication, recent pregnancy or delivery, or obstetric events.

### 2.2 Physical Examination

The neurological examination was significant for right side weakness and left side facial palsy with intact sensation, coordination. The skin and musculoskeletal examinations were unremarkable, apart from hyperpigmentation on her face. No joint deformities or ulcers were noticed.

## 3 Methods

Blood work‐up and basic metabolic panel were unremarkable apart from low potassium and high LDL (Table 1). The immunological workup was positive for B2‐glycoprotein anti‐cardiolipin and lupus anticoagulant. The ANA profile was borderline for PCNA.

**TABLE 1:** Blood work‐up.

		Normal range
HBG	14.8 g/dL	(11–16)
HCT	45.8%	35–54
MCV	97.3 fL	80–100
MCH	31.4 pg	27–34
MCHC	32.3 g/dL	23–36
Platelets	362 × 10^9^/L	150–450
WBC	6 × 10^9^/L	4–10
Neutrophil	52.8%	50–70
Lymphocyte	41.5%	20–55
ESR	20 mm/1 h	—
Urea	21 mg/dL	15–40 mg/dL
S. creatinine	0.9 mg/dL	
Sodium	135 mmol/L	135–145 mmol/L
Potassium	3.3 mmol/L^a^	3.5–5.5 mmol/L
Lipid profile		
Total cholesterol	191 mg/dL	up to 200
Triglyceride	175 mg/dL	up to 150
HDL	46 mg/dL	< 35 = high risk/> 40 low
LDL	110 mg/dL^a^	> 100

Abbreviations: ESR, erythrocyte sedimentation rate; Hb, hemoglobin; Hct, hematocrit; HDL, high‐density lipoprotein; LDL, low‐density lipoprotein; MCH, mean corpuscular hemoglobin; MCHC, mean corpuscular hemoglobin concentration; MCV, mean corpuscular volume.

^a^ Abnormal.

The thrombophilia work‐up revealed a low free protein S level with normal protein C (Table 2).

**TABLE 2:** Anti‐phospholipid and ANA profile.

		Normal range
Antiphosholipids antibodies: B2‐Glycoprotein Ig AGM	2^a^	< 1 −ve, > 1 + ve
Lupus anticoagulant	Detected^a^	
Anticardiolipin (IgG, IgM, IgA)	6 U/mL^a^	< 1 −ve, > 1 + ve
Protein C	90%	70–130
Free protein S	53%^a^	60–140
ANA profile	Borderline for PCNA^a^	

Abbreviation: PCNA = proliferating cell nuclear antigen.

^a^Abnormal.

**FIGURE 1:**
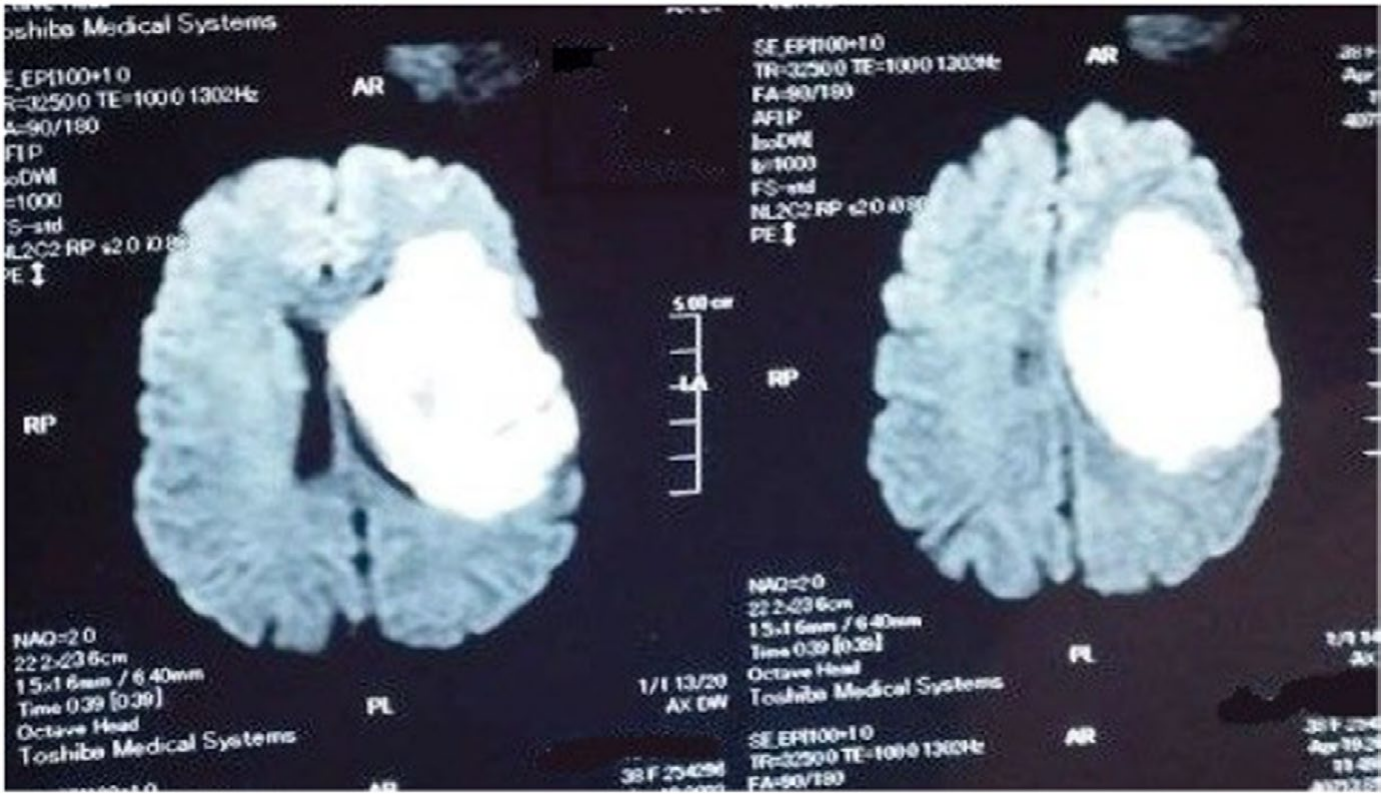
Diffusion‐weighted image showing diffusion restriction.

**FIGURE 2:**
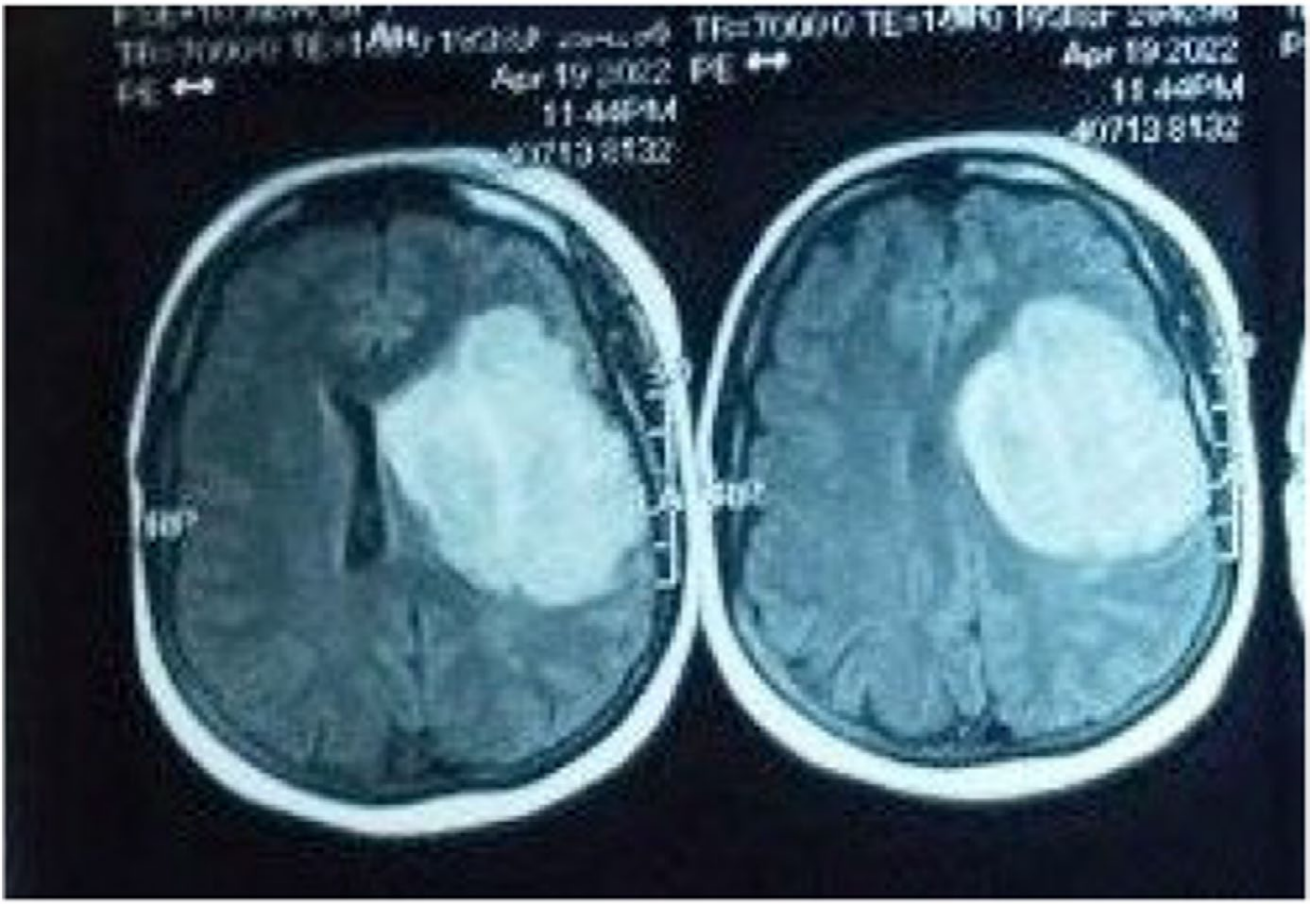
FLAIR showing high signal intensity.

**FIGURE 3:**
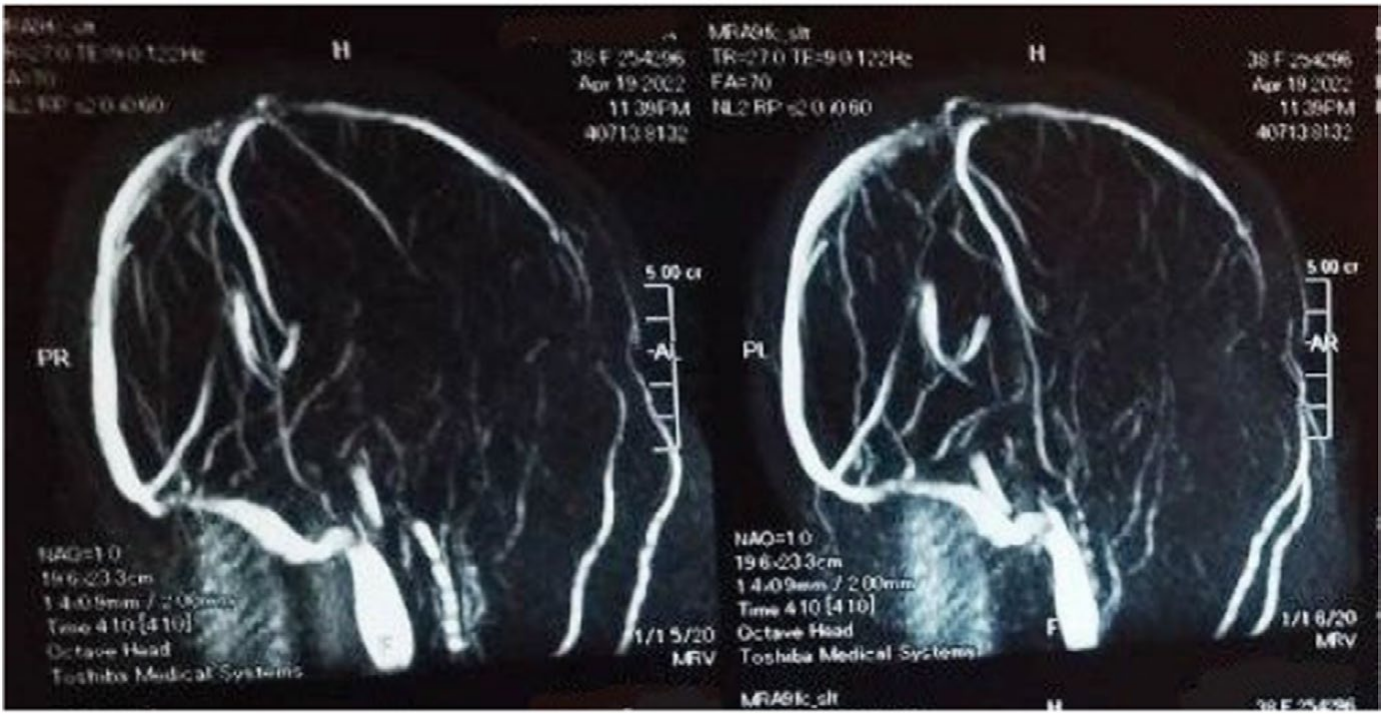
MRV showing loss of signal of left transverse sinus and sigmoid sinus.

Brain CT was unremarkable. MRI revealed left fronto‐pareito‐temporal cortical and subcortical area of cytotoxic edema with internal foci of high signal intensity in T1 representing hemorrhagic transformation with diffusion restriction representing venous infarction (Figures 1 and 2). MRV reports transverse sinus thrombosis with attenuation of the sigmoid and internal jugular veins (Figure 3).

### 3.1 Outcome and Follow Up

As a result of that; diagnosis of CVST has been made. She was started on hydroxychloroquine twice daily, azathioprine, and folic acid in addition to rivaroxaban.

## 4 Discussion

The association between antiphospholipid antibody and protein S deficiency have been widely studied, as this point was a scope of interest for many researchers, for example, the case‐control study that was conducted by M. A. Crowther and colleagues in the late nineties of the previous century revealed that there is an established association between antiphospholipid antibodies and the deficiency of protein S, Crowther has directly suggested that the reason behind APS increased clotting diathesis is the reduced levels of protein s that came as an outcome of APS [11], another study done by PR.AMES since the late nineties also found that there was an accelerated thrombin generation and fibrin turnover in patients who were positive for APS antibodies in both symptomatic and asymptomatic carriers of the illness [12].

R. R. Forastiero studied the pathophysiology behind deficient protein S in patients with APS in 1996 and found that patients with APS who were anticardiolipin positive were found to develop acquired protein S and C4b‐BP deficiency, and those who were lupus anticoagulant positive and anticardiolipin antibody negative were found to develop free protein S deficiency, probably due to the abnormal binding site of free protein S to the C4b‐BP [13]. Connecting all these dots, we find that the association between the APS and free protein deficiency is ubiquitous and not very uncommon.

On the other hand, in the literature review, CVT was mentioned as an uncommon cause of stroke, comprising (0.5%–3%) of total stroke etiology. CVT is more common in females in comparison to males, with a median age of 37 years, as correlates with our patient. According to a study done in India, it was found that among the multiple factors that contributed to CVT, procoagulant factors were the main reason, representing 62.8% of total CVT cases [14]; among others, so many factors were found to cause CVT, such as female‐specific factors, infection, malignancies, lifestyle‐related factors, and inflammatory bowel disease [15].

Among the studies that have been conducted, the relationship between APS and protein S deficiency in most of the cases presented with the manifestation of arterial stroke, for example among the pediatric group a case report in Chilie documented a case of a 12‐year‐old female patient with repeated cerebral infarctions that presented with spastic quadriparesis, she was treated with aspirin and prednisone for 8 months after which her case remitted, it was found that protein S deficiency along with exposure to antiphospholipid might predispose the patient to cerebral infarction [16], another two cases reported in Florida USA among the pediatric group were found to have both APS and protein S deficiency presented in the setting of CVT and coumarin skin necrosis, unlike our patient that presented only with neurological manifestations [17].

Among the adult population, one case report has documented simultaneous occurrence of primary APS and protein S deficiency in a Hispanic male with an ischemic stroke. Unlike our study, there was no noticeable history of CVT [18]. A correlation between APS and protein S deficiency was also identified in a 23‐year‐old female in New York who presented with widespread cutaneous necrosis in the setting of other disorders like SLE and no neurological involvement [19]. The last study we found that documented the association of the two illnesses was a case report revealing the presence of multiple prothrombotic disorders, including APS, protein S deficiency, homocysteinemia, and factor V deficiency, which presented as cutaneous necrosis with no signs of neurological involvement [20].

Our case revealed unremarkable CT report. This is consistent with the usual pattern as only one third of CVT have direct sign [21].

On the other hand MRI may reveal sign of venous congestion and diffusion restriction which was obvious in FLAIR and DWI, respectively [21].

To the best of our knowledge, APS and protein deficiency were never reported in association with both arterial stroke and CVT in adult patients.

## 5 Conclusion

APS has shown an increased predilection to decrease free protein, both reinforcing the chances of developing thrombosis. When presenting together, their most common manifestations are cerebral stroke, cutaneous thrombosis, and CVT. We report a very unusual case of APS and protein S deficiency presenting with cerebral thrombosis and transverse sinus thrombosis, stressing the importance of increasing the index of suspicion for protein S deficiency in antiphospholipid patients who present with cerebral strokes and/or CVT in a young patient.

### 5.1 Imitation

Due to losing follow‐up second work‐up for anti‐phospholipid antibodies was not done. However single test is reasonable because negative patient's drug history for warfarin and other drugs that may cause false positive results and absence of clinical or lab evidence of infection. In addition to that, single positive (SP‐APS) and seronegative APS (SN‐APS) has been described in study done at 2022 by Da Rosa et al. [22]. Positive ANA were more common in SN‐APS/SP‐APS than control APS, which was positive in our study. This study reports that SP‐APS associated with more frequent CVT.

## Author Contributions

**Ziryab Imad Taha Mahmoud:** conceptualization, resources, software, supervision, writing – original draft, writing – review and editing. **Yassin Abdelrahim Abdalla:** conceptualization, project administration, software, supervision, validation, writing – original draft, writing – review and editing. **Hoyam Bakri Gafar Elhaj Omer:** conceptualization, resources, software, validation, writing – original draft, writing – review and editing. **Obada Mohamed Ahmed Ali:** conceptualization, validation, writing – original draft. **Muhammad Salah Ali Suliman:** conceptualization, project administration, writing – original draft. **Asma Awad:** conceptualization, investigation, writing – original draft. **Salih Boushra Hamza:** project administration, supervision, writing – original draft. **Sara Omar Elamin Elmobark:** writing – review and editing. **Mohammedelmuntaga Gafar:** project administration, writing – review and editing. **Abuobieda Omer Osman:** visualization, writing – original draft.

## Consent

Written informed consent was obtained from the patient to publish this case report, and the patient has been informed that their identity will be kept anonymous. The authors have anonymized the case report to protect the patient's privacy. All authors have made substantial contributions to the work and approved the final version of the manuscript.

## Conflicts of Interest

The authors declare no conflicts of interest.

## Data Availability

The data that support the findings of this study are available on request from the corresponding author. The data are not publicly available due to privacy or ethical restrictions.
